# The aggregate-forming pili (AFP) mediates the aggregative adherence of a hybrid-pathogenic *Escherichia coli* (UPEC/EAEC) isolated from a urinary tract infection

**DOI:** 10.1080/21505594.2021.2007645

**Published:** 2021-12-20

**Authors:** Paulo A. Schüroff, Fábia A. Salvador, Cecilia M. Abe, Haleluya T. Wami, Eneas Carvalho, Rodrigo T. Hernandes, Ulrich Dobrindt, Tânia A. T. Gomes, Waldir P. Elias

**Affiliations:** a Laboratório de Bacteriologia, Instituto Butantan, São Paulo, Brazil; b Institute of Hygiene, University of Münster, Münster, Germany; c Departamento de Microbiologia, Imunologia e Parasitologia, Escola Paulista de Medicina, Universidade Federal de São Paulo, São Paulo, Brazil; d Instituto de Biociências, Universidade Estadual Paulista (UNESP), Botucatu, Brazil

**Keywords:** UTI, UPEC, EAEC, hybrid-pathogenic *E. coli*, aggregate-forming pilus

## Abstract

Enteroaggregative *Escherichia coli* (EAEC) comprises an important diarrheagenic pathotype, while uropathogenic *E. coli* (UPEC) is the most important agent of urinary tract infection (UTI). Recently, EAEC virulence factors have been detected in *E. coli* strains causing UTI, showing the importance of these hybrid-pathogenic strains. Previously, we detected an *E. coli* strain isolated from UTI (UPEC-46) presenting characteristics of EAEC, *e.g*., the aggregative adherence (AA) pattern and EAEC-associated genes (*aatA, aap*, and *pet*). In this current study, we analyzed the whole genomic sequence of UPEC-46 and characterized some phenotypic traits. The AA phenotype was observed in cell lineages of urinary and intestinal origin. The production of curli, cellulose, bacteriocins, and Pet toxin was detected. Additionally, UPEC-46 was not capable of forming biofilm using different culture media and human urine. The genome sequence analysis showed that this strain belongs to serotype O166:H12, ST10, and phylogroup A, harbors the *tet, aadA*, and *dfrA*/*sul* resistance genes, and is phylogenetically more related to EAEC strains isolated from human feces. UPEC-46 harbors three plasmids. Plasmid p46-1 (~135 kb) carries some EAEC marker genes and those encoding the aggregate-forming pili (AFP) and its regulator (*afpR*). A mutation in *afpA* (encoding the AFP major pilin) led to the loss of pilin production and assembly, and notably, a strongly reduced adhesion to epithelial cells. In summary, the genetic background and phenotypic traits analyzed suggest that UPEC-46 is a hybrid strain (UPEC/EAEC) and highlights the importance of AFP adhesin in the adherence to colorectal and bladder cell lines.

## Introduction

*Escherichia coli* is a highly versatile Gram-negative microorganism that colonizes the gastrointestinal tract of humans and various animal species. *E. coli* usually comprises an important commensal bacterial of the normal intestinal microbiota. However, according to their set of virulence factors and clinical properties, *E. coli* strains can be classified as diarrheagenic *E. coli*, also designated intestinal pathogenic *E. coli* (IPEC), which are capable of causing diarrhea; and extraintestinal pathogenic *E. coli* (ExPEC), which cause extraintestinal infections, *i.e*., urinary tract infection (UTI), sepsis and meningitis [1–3].

Enteroaggregative *E. coli* (EAEC) represents an important pathotype that belongs to the IPEC group, which is responsible for cases of acute and persistent diarrheal illness in developing countries and cases of travelers´ diarrhea [4–7]. EAEC strains are identified by the production of the aggregative adherence (AA) on cultured epithelial cells, which is characterized by adhered bacteria on the surface of epithelial cells and the coverslip between cells in an arrangement resembling stacked bricks [8,9]. Several virulence factors were identified in the prototypical EAEC 042 strain, which caused diarrhea in volunteers [10]. Among these virulence factors are fimbriae and secreted proteins, such as the aggregative adherence fimbriae (AAF), the anti-aggregation protein (dispersin), the plasmid-encoded toxin (Pet), the protein involved in colonization (Pic), and a pathogenicity island encoding a type 6 secretion system (T6SS) [11–19]. Some of these factors are under the regulation of AggR, an AraC transcriptional activator [20,21].

UTIs are among the most prevalent extraintestinal infections in humans worldwide [22]. Uropathogenic *E. coli* (UPEC) represent the leading cause of community-acquired UTIs (about 70–90%). UPEC infections are highly prevalent in women, children, the elderly, and immunocompromised patients [23,24]. UPEC strains comprehend a heterogeneous pathotype with a wide range of virulence factors that can be combined into different genotypes. Although the isolation source (UTI) implies pathogenicity on epidemiological grounds, different virulence markers have been reported to differentiate UPEC strains from commensal and IPEC strains [25–27]. In addition, these virulence genes play an essential role in ascending UTI promoting the colonization of the bladder and kidney, which can lead to a bloodstream infection, known as urosepsis [22].

In the last years, EAEC has emerged as a causative agent of extraintestinal infections [28–36]. Some studies have described EAEC-associated characteristics, such as the AA pattern on epithelial cells and/or the presence of EAEC-associated genes in collections of *E. coli* strains causing UTI [31,33–37], suggesting that some EAEC strains might have uropathogenic potential. Accordingly, a multiresistant clonal EAEC strain (serotype O78:H10) was associated with an outbreak of UTI in a cluster of 18 patients in Denmark [38,39].

Abe et al. [40] reported IPEC and ExPEC genes in a collection of UPEC strains isolated from patients presenting symptomatic UTI in Brazil. Among the UPEC strains harboring IPEC genetic markers, the strain UPEC-46 presented characteristics of the EAEC pathotype, producing the AA pattern on HeLa cells and harboring the EAEC-associated genes *aatA, aap*, and *pet*. Recently, Nunes et al. [41] showed that UPEC-46 belongs to phylogroup A related cluster composed by the diarrheagenic strain EAEC 17–2 (O3:H2) that presented some ExPEC characteristics [18,42] and the strain EAEC C555-91, which was isolated from a UTI outbreak in Denmark [39]. Santos et al. [43] adopted the terms “hybrid-pathogenic” or “hybrid-pathogen” for strains that harbor defining IPEC and ExPEC virulence factors or are isolated from an extraintestinal infection and exhibit IPEC defining virulence factors. Although hybrid-pathogenic *E. coli* strains have been found in UTI, many questions about the role of these genes in the colonization, virulence potential, and pathogenicity in the urinary tract remain unanswered. Taking these into consideration, UPEC-46 could be classified as a hybrid UPEC/EAEC.

A general analysis of virulence and phenotypic characteristics of the UPEC-46 strain is essential to understand infections caused by hybrid-pathogenic *E. coli* strains. Thus, the goal of this study was the characterization of UPEC-46 by means of phenotypic and genotypic assays and comparative genomic analysis.

## Material and methods

### Bacterial strains

The *E. coli* strain UPEC-46 used in this study was obtained from a patient presenting symptomatic UTI, who was admitted to the emergency room of Hospital São Paulo (a tertiary university hospital in São Paulo city, São Paulo, Brazil). This strain was isolated in pure culture and identified in the Microbiology Service of the Central Laboratory at Hospital São Paulo [40]. The UPEC-46 strain was stored in lysogeny broth (LB) (Difco, USA) supplemented with 15% glycerol at −80°C.

The strains used as controls in various experiments in this study are presented in Supplementary Table S1.

### Antimicrobial resistance profile

The disc diffusion test was performed with Mueller Hinton agar and paper discs (Sensibiodisc – CECON, Brazil) following the standard disk diffusion method recommended by the Clinical and Laboratory Standards Institute [44]. The test discs were tetracycline, gentamicin, ampicillin, streptomycin, kanamycin, chloramphenicol, rifampicin, imipenem, sulfatrim, amikacin, ceftazidime, norfloxacin, ceftriaxone, cefotaxime, cefepime, and cephalothin.

### Adherence assays

HeLa (ATCC CCL-2), HT-29 (ATCC HTB-38), and 5637 (ATCC HTB-9) cells were used to evaluate the ability of the bacterial strain to interact with eukaryotic cells in culture. The qualitative adherence assays were performed as described by Cravioto et al. [45], with some modifications. Briefly, the specific cell lineages were seeded at about 1 × 10^5^ cells per well in 24-well culture plates (Corning, USA) containing 13 mm round glass coverslips. Adherence assays were performed in Dulbecco modified Eagle medium (DMEM) (Cultilab, Brazil) for HeLa/HT-29 cells or Roswell Park Memorial Institute (RPMI) (Cultilab) for 5637 cells, containing 1% D-mannose and 2% fetal bovine serum, and a bacterial inoculum containing bacteria with multiplicity of infection (MOI) of 100. The assay mixture was incubated for 3 h or 6 h at 37°C in 5% CO_2_. After the incubation period, preparations were washed with phosphate-buffered saline (PBS), fixed with methanol, stained with May-Grunwald/Giemsa (Merck Millipore, USA), and examined by light microscopy.

Quantitative adhesion assays were also performed with HeLa, HT-29, and 5637 cells cultivated in 24-well culture plates without coverslips, as described above. After the incubation period (3 h or 6 h), the epithelial cells were lysed in PBS plus 1% (v/v) Triton X-100 for 10 min. Recovered bacteria were serially diluted and plated on MacConkey (MC) agar (Difco) plates, incubated overnight, and the colonies were counted to calculate the CFU/mL. Assays were performed three times in duplicate.

### Biofilm formation assays

The UPEC-46 strain was evaluated for the capacity to form biofilm on glass or polystyrene under the following culture conditions: DMEM high glucose (Cultilab), preconditioned DMEM in HeLa cells, or pooled human urine. Preconditioned DMEM was prepared according to Munhoz et al. [46], using supernatants of HeLa cells cultures in DMEM. Pooled human urine was composed of samples collected from 10 healthy adult female volunteers who had no history of UTI or antibiotic use during 30 days prior to the sample collection. This protocol was approved by the National Council of Ethics in Research of Brazil (CONEP, Ministry of Health, Brazil) under Protocol No. 11159019.7.0000.5474. Urine samples collected from these individuals were pooled, filter sterilized, and stored at −20°C.

All biofilm assays were conducted in the presence and absence of 1% methyl α-D-mannopyranoside (α-D-man) (Sigma-Aldrich, USA) and performed three times in triplicate. The biofilm assays in polystyrene and glass surfaces were performed following the protocols previously described [47,48]. For the kinetics of biofilm formation, overnight bacterial cultures grown in LB were inoculated in a 1:40 ratio under five incubation intervals (3, 6, 9, 12 or 24 h). In these experiments, the cutoff (forming and non-forming biofilm) for the UPEC-46 strain was established according to Stepanovic et al. [49], using the *E. coli* strain DH5α as negative control.

### Curli, cellulose and bacteriocin production

The production of curli fimbriae was evaluated using Congo red agar plates incubated at 26°C during 48 h or at 37°C during 24 h [50]. Cellulose production was detected on cellulose agar plates, as previously described [51]. The plates were also incubated at 26°C during 48 h or 37°C during 24 h. The bacteriocin production assay was based on the method described by Pugsley and Oudega [52].

### Detection of Pet in culture supernatant

The secretion of Pet in the culture supernatant of UPEC-46 was investigated by immunoblotting using a rabbit polyclonal anti-Pet serum (anti-Pet IgG) [53].

### Plasmid DNA analyses

The plasmid profile of UPEC-46 was determined using the protocol of Birnboim and Doly [54], and the plasmid content of *E. coli* strain 39R861 [55] was used as a molecular size marker and control of extraction. The conjugation experiments were based on the protocol previously described [56]. UPEC-46 (tetracycline-resistant) was used as the donor strain and *E. coli* MA3456 (nalidixic acid-resistant) as the recipient.

### Whole-genome sequencing

Long-read Oxford Nanopore MinION (Oxford Nanopore, UK) and short-read Illumina Hiseq 1500 (Illumina, USA) platforms were combined to generate the whole-genome sequence. To generate short-read sequences, the rapid protocol (2x250 paired-end reads) was performed according to the manufacturer’s protocol for sequencing. For long-read genome sequencing, a MinION sequencing library was prepared using the Nanopore Ligation Sequencing Kit (Oxford Nanopore). The library was sequenced with an R9.4.1 MinION flow cell for a 24 h run using MinKNOW (v2.0) with the default settings. The FAST5 files were base called and converted to FASTQ format in real-time using Guppy (v3.3.0, Oxford Nanopore). Prior to assembly, the raw reads were quality checked with FastQC (v0.11.5, http://www.bioinformatics.babraham.ac.uk/projects/fastqc), and low-quality reads were trimmed using Sickle (v1.33, http://github.com/najoshi/sickle). Long reads were then filtered by quality using Filtlong (v0.2.0) program, available at http://github.com/rrwick/Filtlong.

### *Genome assembly, annotation, and* in silico *analyses*

Hybrid de novo genome assembly of the Nanopore and Illumina reads was performed using Unicycler (v0.4.8) [57], with default parameters. The genome assembly was analyzed using QUAST (v4.3) [58], and contigs smaller than 500 bp were discarded. The DNAPlotter (v18.0.0) [59] was used to generate circular images of plasmid sequences. Moreover, Prokka (v1.12) [60] was used to determine coding sequences (CDS) and to automatically annotate the genome. The different web-based databases ClermonTyping (v1.4.1) [61], MLST (v2.0) [62], PlasmidFinder (v2.1) [63], ResFinder (v3.2) [64], and SerotypeFinder (v2.0) [65] were used to determine *in silico* the *E. coli* phylogroups, sequence type, plasmids, acquired resistance genes, and serotypes, respectively.

All raw sequencing data generated for this study are deposited in the NCBI sequence read archive (SRA) under the accession number PRJNA728080. The whole-genome sequences (WGS) of UPEC-46 were deposited in the GenBank database under the accession number JAHBCK000000000. The plasmid sequences p46-1, p46-2, and p46-3 replicons were deposited in the GenBank database under the accession numbers NZ_JAHBCK010000003, NZ_JAHBCK010000004, and NZ_JAHBCK010000007, respectively.

### Phylogenetic analyses

The different phylogenetic trees were constructed using single nucleotide polymorphism (SNP)-based phylogeny employing the draft genome sequence of strain UPEC-46 and selected reference strains. The SNP-based phylogenetic trees were created using different core genome alignments present in the parameter settings of Snippy (v3.1.0), available at https://github.com/tseemann/snippy. Recombinant genomic regions were removed using Gubbins (v2.20) [66] and the maximum-likelihood (ML) phylogenetic trees were inferred using the RAxML (v 8.2.12) [67] GTRGAMMA model with 1,000 bootstrap resampling. Finally, the trees were visualized with iTOL (v4) [68].

### Identification of virulence-associated genes in strains harboring the aggregate-forming pili (AFP)-encoding genes

The *afp* operon was searched in the genome assembly and annotation reports of 18,360 *E. coli* genomes deposited in the GenBank database (accessed on 20 November 2019). The presence of virulence factors was searched using distinct *E. coli* virulence factor groups with 1,154 deduced protein sequences of virulence-associated genes [69].

An automated search for protein homologs in annotated bacterial genomes and the detection of *E. coli* virulence factors were performed with the “prot_finder” pipeline of the “bac-genomics scripts” collection using BLASTP+ (v2.2) [70]. Additionally, a phylogenetic tree with AFP-positive strains was created as described above and combined with the virulence genes panel. The heatmap of the corresponding binary presence/absence matrix was generated using the online tool iTOL.

### Construction and analysis of genetically modified strains

The plasmids and strains used for the construction of mutants and complemented strains are described in Supplementary Table S1.

A nonpolar mutation in *afpA*, the major structural subunit of AFP, was constructed in UPEC-46 using the suicide vector pJP5603 [71]. Briefly, a 307-bp DNA fragment corresponding to the internal region of *afpA* was amplified with primers 4532-Fw (5′-AACAAGACTCAGAGCACCGT-3′) and 4532-Rv (5′-CATTAACCCCGACACCACC-3′) and cloned into the pGEM-T Easy vector (Promega, USA). This construction was digested with *EcoRI*, and the insert was subcloned in pJP5603, generating pPAS2, which was transformed into *E. coli* S17-1(λ*pir*) cells [72]. One transformant was used to mobilize pPAS2 into strain UPEC-46 [73]. Transconjugants were selected on LB agar plates containing kanamycin (50 µg/mL) and tetracycline (25 µg/mL). The correct site of integration was confirmed by Sanger sequencing using the ABI 3730 DNA Analyzer system (ThermoFisher, USA), and the mutant was named as UPEC-46::*afpA.*

For complementation purpose, the entire *afpA* gene was amplified from UPEC-46 by PCR using the primers FwAfpA (5′-AATGCTCGAGATGAATATTTTTACAAAAAAAG-3′) and RvAfpA (5′-TCACAAGCTTTTATTTCAGCAGGAAGGT-3′) and the amplicon was cloned into pACYC177 [74], generating pPAS3. Sanger sequencing was used to confirm the correct insertion and absence of mutation in the *afpA* gene, and UPEC-46::*afpA* was transformed with pPAS3 generating UPEC-46::*afpA*(pPAS3).

UPEC-46 and derivative strains were phenotypically analyzed. Single colonies were inoculated in 3 mL of LB and incubated overnight at 37°C. Each culture was then sub-cultured into 50 mL of LB, DMEM, or RPMI medium in a 1:100 ratio and incubated at 37°C with shaking. The optical density (OD) was monitored at 30 min intervals for 6 h by spectrophotometry (600 nm). This test was performed in triplicates. Strains were also evaluated on motility agar (LB containing 0.3% agar). After incubation at 37°C for 18 h, the halos obtained were measured. This test was performed three times in duplicate.

### Production of anti-AFP polyclonal serum

Anti-AFP polyclonal serum was obtained in a female New Zealand white rabbit as described by Evans et al. [75], with some modifications. The protocol was approved by the Ethics Committee on Animal Use of the Butantan Institute (CEUAIB Protocol No. 7146050620).

Initially, a single colony of UPEC-46 was inoculated in 3 mL of LB and incubated at 37°C for 18 h statically. Then, the bacterial culture was centrifuged at 700 x g for 10 min, the pellet was resuspended in 0.5% formalin in PBS and diluted to approximately 3 × 10^8^ CFU/mL. The rabbit was inoculated intravenously at 3-days intervals with increasing volumes from 0.5 to 4.0 mL. The rabbit was bled 14 days after the eighth dose. The obtained serum was then adsorbed with the AFP-mutant strain (UPEC-46::*afpA*).

The pre-immune and anti-AFP sera were analyzed by immunoblotting against UPEC-46 and UPEC-46::*afpA*. Briefly, the wild-type and the AFP mutant strains were incubated statically in 1 mL of LB at 37°C for 18 h. Subsequently, the cells were pelleted, resuspended in 200 μL of PBS, and analyzed by SDS-PAGE (12%) [76]. Separated proteins were transferred to nitrocellulose membranes that were immunodetected with either pre-immune or anti-AFP sera as the primary antibody (1:500) and the goat anti-rabbit IgG (1:5,000) as the secondary antibody. The membranes were revealed by ECL chemiluminescence system (Amersham, USA), according to the manufacturer’s instructions.

### Immunogold labeling of AFP

The immunogold labeling of AFP from UPEC-46 and derivative strains were performed using pre-immune or anti-AFP sera (1:10), as primary antibodies, and 1:10 diluted goat anti-rabbit IgG conjugated with 10 nm colloidal gold (Sigma-Aldrich). Preparations were negatively stained with 2% uranyl acetate on Formvar-coated nickel grids and examined under transmission electron microscope (LEO 906E – Zeiss, Germany), operated at 80 kV [46].

### Statistical analyses

Statistical analyses were conducted using the GraphPad Software package (v7.0, GraphPad Software, USA). Results were analyzed by one-way analyses of variance (ANOVA) followed by Dunnett’s multiple-comparison test or Tukey’s multiple-comparison test. The mean values ± standard deviations (SD) are shown in the figures and statistical significance was established at p < 0.05.

## Results

### UPEC-46 is phylogenetically related to EAEC strains with a similar virulence profile

The whole genome of UPEC-46 was sequenced, generating 23 contigs (≥ 500 bp), where three were closed as plasmids with different sizes: 135,351 bp, 108,910 bp, and 9,161 bp. The predicted genome size was 5,087,484 bp, with 50.65% of GC content.

The *in silico* serotyping was performed by analyzing the *wzx* and *wzy* gene sequences, identifying the serogroup O166. The *fliC* gene was assigned to the H12 type. Therefore, the UPEC-46 strain belongs to serotype O166:H12. Upon automatic annotation with Prokka, 4,807 CDS, 4,902 genes, and 94 tRNA were identified. Different virulence factors were recognized to be encoded by the annotated CDSs, such as adhesins, invasins, iron uptake systems, bacteriocins, toxins, and genes involved with serum resistance (Table 1).

**Table 1.** Virulence factors identified in UPEC-46

Virulence traits	Virulence factor	Name	Associated with
Adhesion/Invasion	Aap	Dispersin	IPEC
	Aat	Anti-aggregative transporter^1^	IPEC
	Csg	Curli^1^	Various
	IbeB	Invasion protein	ExPEC
	IbeC	Invasion protein	ExPEC
	NlpI	Lipoprotein	ExPEC
Bacteriocin	ColE1	Colicin E1	ExPEC
	Mcb	Microcin B17^1^	ExPEC
CU fimbriae	Ecp	*E. coli* common pilus^1^	Various
	Fim	Type 1 fimbriae^1^	Various
	Sfm	Sfm fimbriae^1^	Various
	Yad	Yad fimbriae^1^	Various
	Ybg	Ybg fimbriae^1^	Various
	Ycb	Ycb fimbriae^1^	Various
	Yde	Yde fimbriae^1^	Various
	Yeh	Yeh fimbriae^1^	Various
	Yfc	Yfc fimbriae^1^	Various
	Yhc	Yhc fimbriae^1^	Various
	Yra	Yra fimbriae^1^	Various
Iron uptake	Efe	Efe system^1^	Various
	Ent, Fep	Enterobactin^1^	Various
	Fec	Ferric citrate transport^1^	Various
	Feo	Transport of ferrous^1^	Various
	Fhu	Ferrichrome uptake^1^	Various
	Ybt, Irp2, Irp1	Yersiniabactin biosynthetic system^1^	Various
	FyuA	Yersiniabactin siderophore receptor	ExPEC
Serum resistance	Iss	Serum survival	ExPEC
	Etk, Etp, Gfc	Group 4 capsule^1^	ExPEC
T2SS	Gsp	T2SS-1^1^	Various
T3SS	Eiv, Epa, Epr, Yge	ETT2^2^	Various
T4P	AFP	Aggregate-forming pili^1^	IPEC
T5SS	AatA	APEC autotransporter	ExPEC
	Pet	Plasmid-encoded toxin	IPEC
	UpaC	UPEC autotransporter C	ExPEC
	UpaI	UPEC autotransporter I	ExPEC
	EhaC	EHEC autotransporter C	IPEC
T6SS	Aai	AggR-activated island^2^	IPEC
Toxins	AstA	EAST1 toxin	IPEC
	HlyE	Hemolysin E	Various

1, operon complete; 2, operon incomplete; CU, chaperone usher; T2SS, type 2 secretion system; T3SS, type 3 secretion system; T4P, type 4 pili; T5SS, type 5 secretion system; T6SS, type 6 secretion system.

We also evaluated the uropathogenic potential based on the presence of the virulence markers *chuA* (heme receptor), *fyuA* (yersiniabactin siderophore receptor), *vat* (vacuolating autotransporter protein), and *yfcV* (YfC fimbriae), as defined by Spurbeck et al. [27]. UPEC-46 strain did not meet this criterion since only *fyuA* and *yfcV* were detected. Interestingly, none of the genes defined by Johnson et al. [77], as markers of ExPEC intrinsic virulence potential (*papA* and/or *papC, afa/dra, sfa/foc, iucD/iutA*, and *kps*MT II) were found in the genome of UPEC-46.

**Figure 1.**
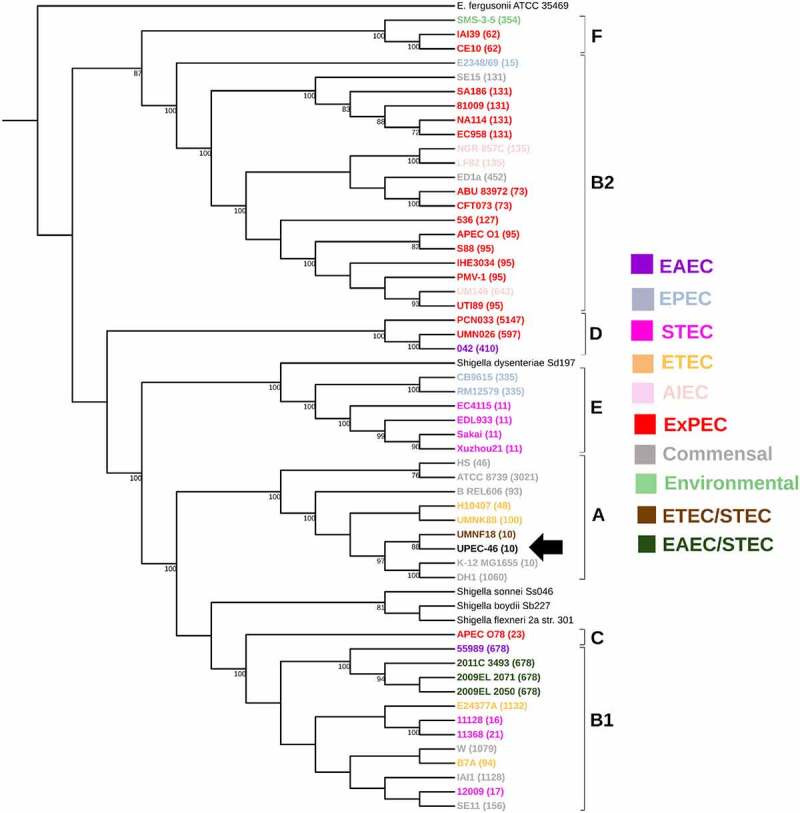
Whole genome-based phylogenetic of UPEC-46 and reference *E. coli* strains. The maximum-likelihood phylogenetic tree was constructed with 1,000 bootstrap replicates. The tree was visualized with iTOL and *E. coli* pathotypes or *E. coli* groups are indicated by colors. ST numbers from the MLST analysis for each strain are given in parentheses and phylogroups appointed. The UPEC-46 strain is indicated by a black arrow. *E. fergusonii* serves as an outgroup

In the phylogenetic analyses, firstly, we created a general phylogenetic tree using the whole genome of UPEC-46 strain compared to genomes from 51 reference *E. coli* strains from public databases (Supplementary Table S2). The reference strains comprised different *E. coli* groups, such as commensal, environmental, IPEC, and ExPEC. These groups belong to diverse MLST and phylogenetic lineages (A, B1, B2, C, D, E, and F), indicating a high phylogenetic diversity in the strain panel. The phylogenetic tree constructed presented in general high bootstrap support values, and the UPEC-46 isolate clustered with the strains belonging to ST10 and phylogroup A, as shown in Figure 1.

**Figure 2.**
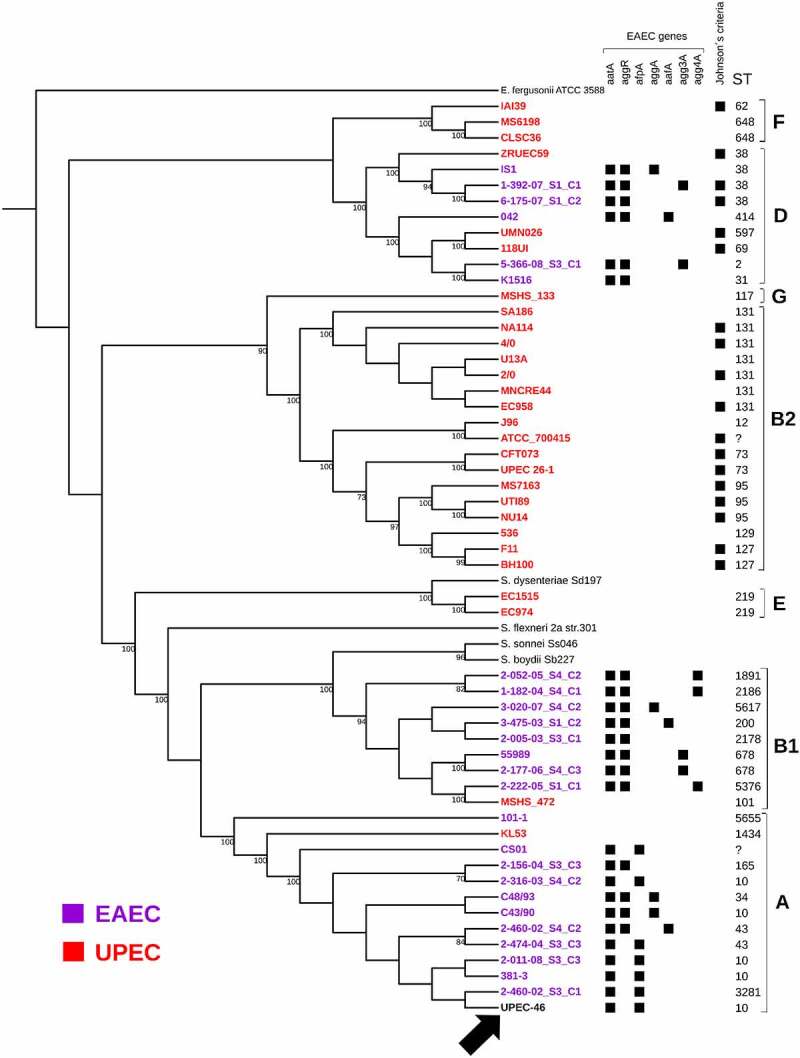
Whole genome-based phylogeny and genetic characteristics of UPEC-46 and selected EAEC and UPEC strains. The maximum-likelihood phylogenetic tree was constructed with 1,000 bootstrap replicates. The tree was visualized with iTOL, where UPEC-46, EAEC, and UPEC strains are indicated by black, purple, and red colors, respectively. The different STs and phylogroups are appointed. The following EAEC-associated virulence genes were searched: *aggR* (virulence regulator), *aatA* (anti-aggregation protein transporter), *aggA* (AAF/I fimbriae), *aafA* (AAF/II fimbriae), *agg3A* (AAF/III fimbriae), *agg4A* (AAF/IV fimbriae), and *afpA* (AFP, type 4 pili). The strains were positive for Johnson’s criteria (criteria for ExPEC) if positive for ≥2 of the five ExPEC markers, i.e., *pap* (P fimbriae), *sfa/foc* (S/F1C fimbriae), *afa/dra* (Dr binding adhesins), *iucD/iutA* (aerobactin receptor), and *kpsMT II* (group 2 capsule synthesis) [77]. The UPEC-46 strain is indicated by a black arrow. *E. fergusonii* serves as an outgroup

With a second phylogenetic analysis, we evaluated the relationship between UPEC-46 and representative EAEC and UPEC strains belonging to diverse phylogenetic and MLST lineages (Supplementary Table S3). As expected, this analysis showed that the majority of UPEC strains were distributed in three phylogenic groups B2, F, and D, whereas EAEC strains belonged to phylogroups A, B1, and D. UPEC-46 was related to EAEC strains since it was located in a clade closely associated only with EAEC strains, which do not meet the Johnson's criteria [77], defining *E. coli* strains with intrinsic ExPEC virulence (Figure 2).

**Figure 3.**
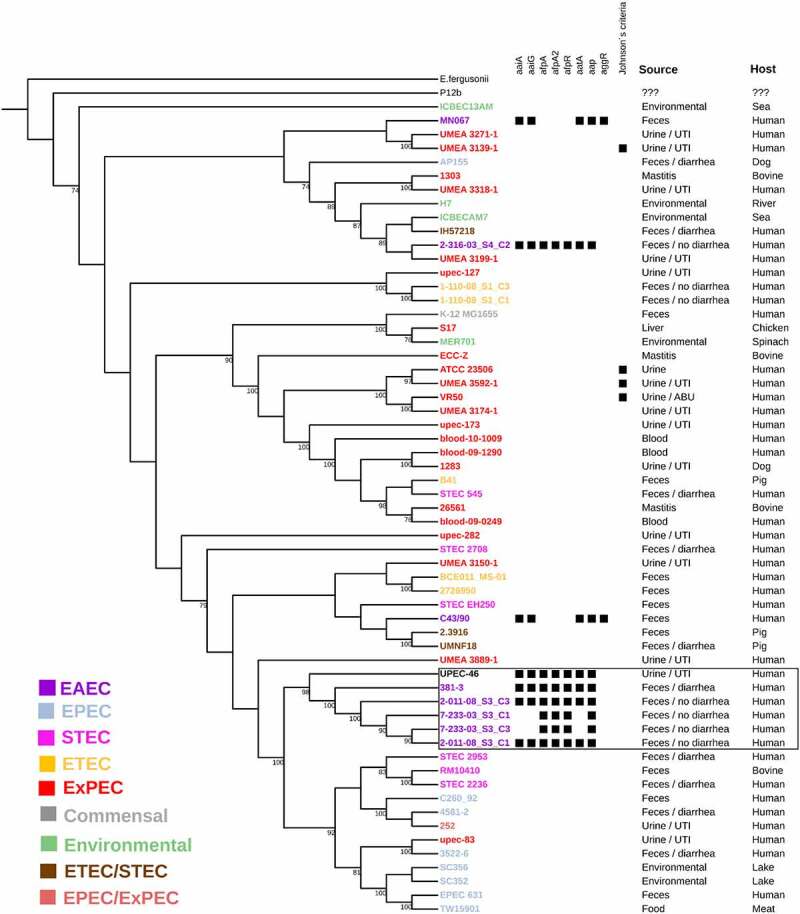
Whole genome-based phylogeny and genetic characteristics of UPEC-46 and selected *E. coli* ST10 strains. The phylogenetic tree was constructed by using the maximum-likelihood method and bootstrap with 1,000 replicates. The tree was visualized with iTOL and different *E. coli* pathotypes or *E. coli* groups are designated with a color code. The following UPEC-46-associated virulence genes were searched: *aggR* (virulence regulator), *aatA* (anti-aggregation protein transporter), *aaiAG* (*aggR*-activated Island), *aap* (dispersin, anti-aggregation protein) and *afpA, A2, R* (AFP, type 4 pili). The strains were positive for Johnson’s criteria (criteria for ExPEC) if positive for ≥2 of the five ExPEC markers, i.e., *pap* (P fimbriae), *sfa/foc* (S/F1C fimbriae), *afa/dra* (Dr binding adhesins), *iucD/iutA* (aerobactin receptor) and *kpsMT II* (group 2 capsule synthesis) [77]. UPEC-46 strain is related to strains with similar virulence profile (*afp* positive) present in the box. *E. fergusonii* serves as outgroup. UTI: urinary tract infection; ABU: asymptomatic bacteriuria

In a third phylogenetic tree, the relationship between UPEC-46 and several ST10 *E. coli* strains, isolated from distinct sources, was assessed. This analysis showed that UPEC-46 is phylogenetically close to a group of EAEC strains with a similar virulence profile, incl. genes associated with the AFP adhesin (*afpA, afpA2* and *afpR*), and other EAEC genes *(aatA, aap, aaiA*, and *aaiG*) [78]. Interestingly, these strains were isolated from human feces as part of the microbiota or involved in diarrheal cases, devoid of *aggR* and negative for the criterion of ExPEC intrinsic virulence [77] (Figure 3 and Supplementary Table S4).

Therefore, by different phylogenetic comparisons, UPEC-46 was phylogenetically related to EAEC strains isolated from human feces, with similar virulence profiles (*aggR*-/*afp*+).

### UPEC-46 harbors three circular plasmids

**Figure 4.**
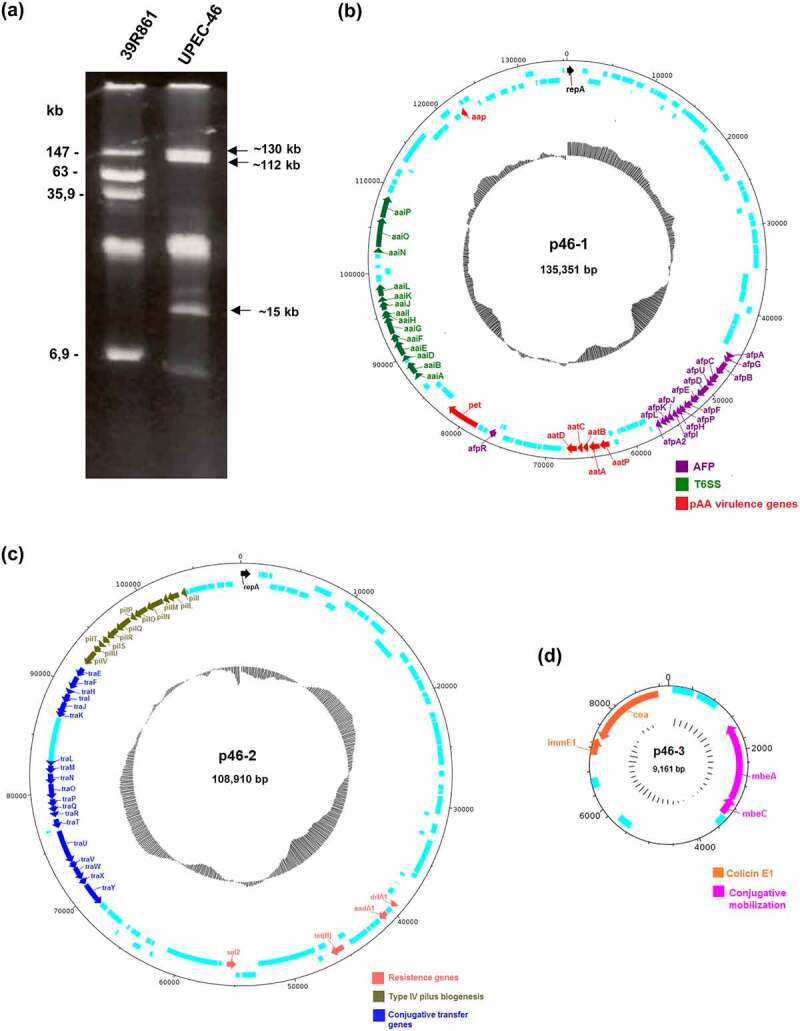
Plasmid profile and comparison of plasmids architecture present in UPEC-46. (a) Plasmid content of the UPEC-46 strain obtained by alkaline extraction, followed by electrophoresis in 0.8% agarose gel in Tris-Borate-EDTA (TBE) buffer. Approximate sizes were predicted based on the plasmid migration in agarose gel. The *E. coli* strain 39R861 represents the standard strain containing plasmids of known molecular weights. (b) Plasmid p46-1 (virulence plasmid) carries genes typical for EAEC strains (*aatA, B, C, D*, and *P; aap; pet*). Additionally, it contains an operon encoding an aggregate-forming pilus (*afp*) and an operon encoding a type 6 secretion system (T6SS, *aai*). (c) Plasmid p46-2 (encoding antibiotic resistance) harbors genes for the pil pilus (type 4 pili biogenesis), conjugative transfer genes and resistance genes. (d) Plasmid p46-3 (colicinogenic plasmid) carries genes for colicin E1 synthesis and conjugative mobilization genes. CDS are presented in light blue and GC-content is depicted in black inner circles

Plasmid profile analyses by agarose gel electrophoresis confirmed that UPEC-46 carries three plasmids: two bands of ~130 kb and ~112 kb indicated plasmids with high molecular size in addition to one plasmid band of approximately ~15 kb (Figure 4(a)).

Analyzing the plasmid sequences, we observed that the ~135 kb plasmid (plasmid p46-1) contains a complete *afp* operon (*afpAGBCUDEFPHIJKLA2*) as well as *afpR* coding for the corresponding AraC-like regulator. The *afp* operon comprises the genes responsible for the biogenesis of aggregate-forming pili (AFP). p46-1 also harbors a truncated putative type 6 secretion system (T6SS) termed as *aaiA-P*, where *aaiC* and *aaiM* are absent. In addition, this plasmid harbors the following EAEC-associated genes: *aatPABCD* operon, *aap*, and *pet* (Figure 4(b)), commonly associated with the pAA plasmid present in the prototypical EAEC strain 042 [79]. The ~109 kb plasmid (plasmid p46-2) carries the resistance genes *aadA1, dfrA1, sul2*, and *tet(B)* and confers resistance to various antibiotic agents, such as streptomycin, trimethoprim, sulfamethoxazole, and tetracycline, respectively. p46-2 carries a type 4 pili biosynthesis locus (*pilI-V*) and the conjugative transfer genes (*tra* operon), associated with bacterial conjugation (Figure 4(c)). p46-3 is the smallest plasmid (~9 kb) harboring the colicin E1 gene (*cea*) and the genes *mbeA* and *mbeC*, associated with conjugative mobilization proteins (Figure 4(d)). Lastly, two different replicon sequences (IncFII and IncB/O/K/Z) were found in UPEC-46 using the program PlasmidFinder, which belong to p46-1 and p46-2, respectively.

To verify if these plasmids could be related to the virulence and antimicrobial resistance profile of UPEC-46, conjugation experiments were performed using strain UPEC-46 as donor and *E. coli* MA3456 as recipient. Among eleven transconjugants obtained, three carried both the ~112 and ~15 kb plasmid (Supplementary Figure S1), whereas eight carried only the ~112 kb plasmid. None of the eleven transconjugants produced the AA pattern on HeLa cells (data not shown) indicating that the AA pattern-related determinants were not located in these plasmids. Then, they were evaluated for their antimicrobial resistance profile. UPEC-46 showed antimicrobial resistance against streptomycin, sulfatrim (combination of sulfamethoxazole/trimethoprim), and tetracycline. Likewise, the eight transconjugants harboring only the ~112 kb plasmid presented resistance to these antibiotics, confirming that the ~112 kb plasmid conjugative plasmid carried the UPEC-46 antimicrobial resistance determinants.

### UPEC-46 has phenotypic characteristics associated with EAEC and ExPEC

**Figure 5.**
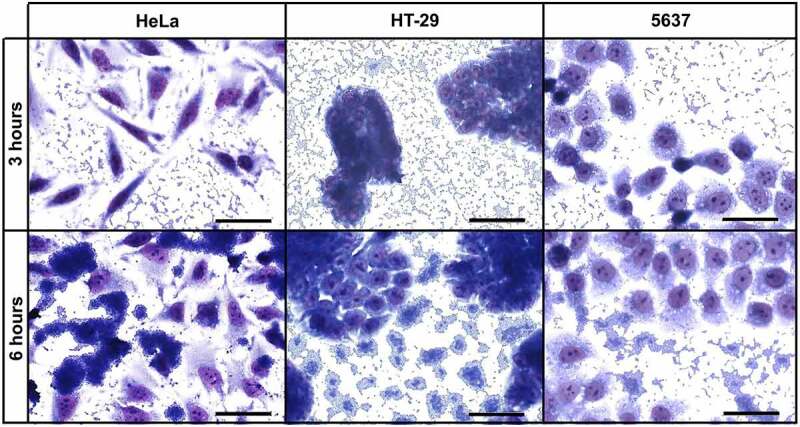
Qualitative adherence assay of UPEC-46 with different cell lineages. The patterns were identified after 3 h and 6 h of infection, in the presence of 1% D-mannose, using HeLa (cervical carcinoma), HT-29 (colorectal adenocarcinoma) and 5637 (human urinary bladder) cells. Evaluation of patterns by light microscopy. Bars = 50 µm

The ability of UPEC-46 to produce the AA pattern on different epithelial cell lineages was investigated. As previously reported [40], this strain adhered to HeLa cells presenting the typical AA pattern, observed after 3 h and more intensely after 6 h of bacteria-HeLa cell interaction. Likewise, UPEC-46 was able to colonize intestinal (HT-29) and urinary bladder epithelial (5637) cells, and the AA pattern was observed on both cell lines after 3 h, and more intensely after 6 h incubation (Figure 5). All adherence assays were performed in the presence of 1% D-mannose to inhibit type 1 fimbria-mediated bacterial adherence.

**Figure 6.**
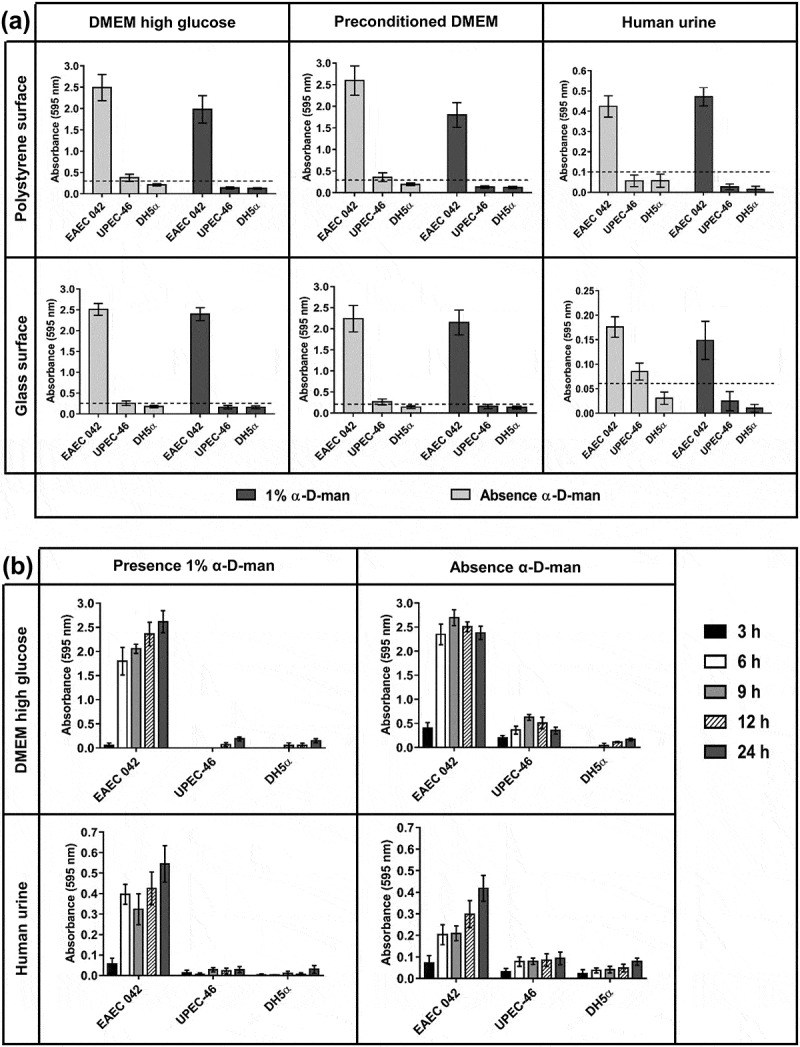
Biofilm and kinetics of biofilm formation of UPEC-46. (a) The biofilm formation assays were performed in different culture media (DMEM high glucose, preconditioned DMEM, and human urine) and abiotic surfaces (polystyrene and glass), with incubation for 24 h at 37°C. The dashed line represents the cutoff OD between forming and non-forming biofilm strains [49]. The cutoff was defined as three standard deviations above the mean OD of the negative control. (b) The kinetic assays were performed in DMEM high glucose or human urine culture media during different incubation periods (3, 6, 9, 12, and 24 h) on polystyrene surface at 37°C. These tests were performed in the presence or absence of 1% α-D-man. In all tests performed, EAEC 042 and *E. coli* DH5α were used as positive and negative controls, respectively. The assays were performed in triplicate and repeated three times. The data presented consist of the mean ± standard deviation

As the AA pattern is strongly associated with biofilm formation on abiotic surfaces [47], UPEC-46 was evaluated for this phenotype using different culture conditions (DMEM high glucose, preconditioned DMEM, and human urine) on glass or polystyrene surfaces. As shown in (Figure 6(a)), after 24 h of incubation in the presence of α-D-man, UPEC-46 could not form biofilm in all conditions tested, showing similar ODs as the negative control (*E. coli* DH5α). In the absence of α-D-man, UPEC-46 biofilm formation was weak and did not markedly differ between the different conditions analyzed, presenting absorbance values lower than the positive control (EAEC 042). As shown in (Figure 6(b)), the kinetics of biofilm formation on polystyrene was also evaluated upon 3, 6, 9, 12, and 24 h of incubation and under different culture conditions (DMEM high glucose vs. human urine). In the presence of α-D-man, UPEC-46 presented low absorbance values, similar to the negative control, in both conditions tested. In the absence of α-D-man, although still lower than the positive control (EAEC 042), the absorbance values for UPEC-46 were higher, increasing until 9 h of incubation, before decreasing again at later time points.

**Figure 7.**
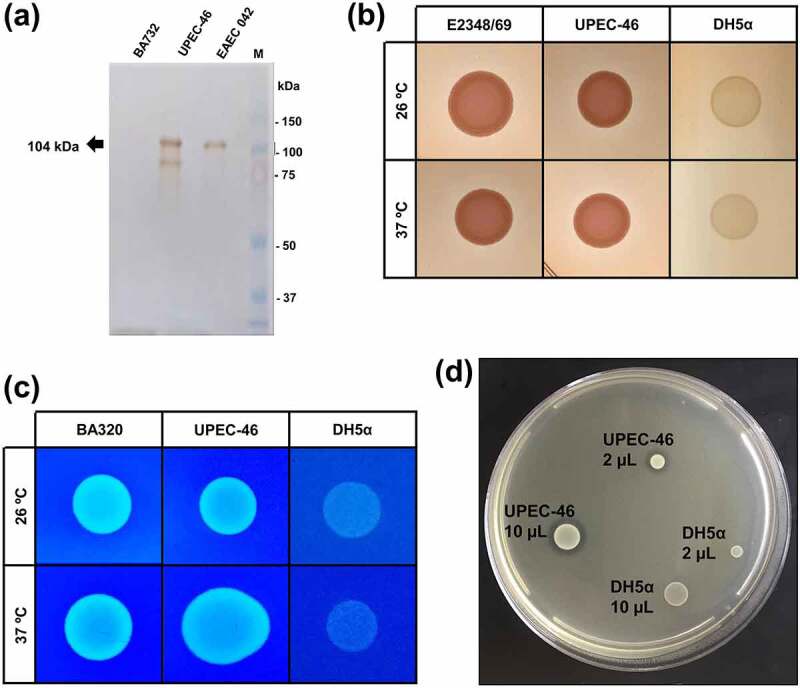
Phenotypic characteristics of UPEC-46. (a) Pet detection in the UPEC-46 using culture supernatants. The bacterial supernatants were cultivated in LB and precipitated with Trichloroacetic acid (TCA). Immunoblotting was performed with anti-Pet IgG and developed with Diaminobenzidine. Positive control: EAEC 042. Negative control: EAEC BA732. M: Precision plus protein™ dual color standards (Bio-Rad, USA) used as molecular weight. (b) Analysis of curli expression using the Congo red agar at 26°C and 37°C. EPEC E2348/69 and *E. coli* DH5α represent positive and negative controls, respectively. (c) Cellulose expression using the cellulose agar at 26°C and 37°C. *E. coli* BA320 and DH5α were used as positive and negative controls, respectively. (d) Bacteriocin production of UPEC-46. A drop of 2 and 10 μL of the overnight culture (UPEC-46 and negative control) was placed on a plate containing a freshly prepared lawn of *E. coli* C600 (indicator strain for bacteriocin production). After overnight incubation at 37°C, the plate was examined for clear zones. *E. coli* DH5α was used as a negative control

As the Pet-encoding gene was detected in UPEC-46 [40], we evaluated the secretion of Pet in the culture supernatant. Using a polyclonal serum against Pet, a 104 kDa protein was recognized in precipitated culture supernatants of UPEC-46 and EAEC 042 (positive control), strongly suggesting that Pet is secreted by UPEC-46 (Figure 7(a)).

Also, other important phenotypic analyses were performed with UPEC-46, including the detection of curli, cellulose, and bacteriocin production. As presented in (Figure 7(b,c)), curli and cellulose expression was detected upon incubation at 26 and 37°C. Bacteriocin production was detected by the presence of a halo surrounding the UPEC-46 colonies resulting from growth inhibition of the indicator strain *E. coli* strain C600 (Figure (7d)).

### *AFP-positive* E. coli *strains exhibit different virulence profiles*

In the phylogenetic analyses we observed the presence of *afp* genes (*afpA, afpA2*, and *afpR*) in EAEC strains closely related with UPEC-46. Since AFP has been recently described [78], there is not much information on the genotypic characteristics of AFP-positive strains and whether there is indeed a functional convergence in terms of virulence genes. In order to characterize AFP-positive EAEC more precisely with regard to their virulence gene pool, all *E. coli* genomes deposited in the NCBI database were searched for the presence of the *afp* gene cluster and the virulence gene profiles of these genomes were determined.

The genome sequence analysis identified 25 *afp*-positive *E. coli* genomes deposited in NCBI (at the date of 20 November 2019) (Supplementary Table S5). The different *in silico* analyses performed with the 25 *afp*-positive strains and UPEC-46, indicated that 24 strains belonged to phylogroup A (including UPEC-46), whereas two strains belonged to phylogroup B1. Ten and six strains were allocated to two major MLST lineages (ST10 and ST43), respectively. The remaining ten strains represented different STs. In addition, all analyzed strains harbored at least one plasmid, and the IncF plasmid replicon was detected in all strains (except for the strain 381–3). Antibiotic resistance genes were predicted in 18 isolates and different resistance profiles were found. We also observed that these strains had different EAEC virulence gene profiles, where the *aatA* gene, described as strictly associated with AFP-positive strains [78,80], was not found in two *E. coli* strains (7–233-03_S3_C1 and 7–233-03_S3_C3). The *in silico* characteristics of the AFP-positive strains are presented in Table 2.

**Table 2.** Genome sequence-based characterization of AFP-positive strains

AFP-positive strain	Phylogroup	Serotype	Sequence type	Plasmid replicon sequences	EAEC-associated genes	Antibiotic resistance genes
2–005-03_S4_C1	A	ONT:H10	43	IncB/O/K/Z, IncFII, IncQ1	*aatA, aap*	*sul1, sul2, aph(3”)-Ib, aph(6)-Id, tet(A), dfrA14, dfrA7, blaTEM-1B*
2–011-08_S3_C1	A	ONT:H10	10	Col, IncFII	*aatA, aap, astA*	*sul2, dfrA8, aph(3”)-Ib, aph(6)-Id, blaTEM-1B*
2–011-08_S3_C2	A	O111:H12	43	ColpVC, IncB/O/K/Z, IncFII, IncQ1	*aatA, aap*	*blaTEM-1B, tet(A), dfrA14, dfrA7, aph(3”)-Ib, aph(6)-Id, sul1, sul2*
2–011-08_S3_C3	A	ONT:H10	10	IncFII	*aatA, aap, astA*	*dfrA8, aph(6)-Id, blaTEM-1B*
2–316-03_S4_C2	A	ONT:H10	10	IncFII	*aatA, aap*	*blaTEM-1B, sul2, dfrA8, aph(3”)-Ib, aph(6)-Id*
2–460-02_S3_C1	A	O111:H12	3281	IncB/O/K/Z, IncFII	*aatA, aap*	*dfrA14, aph(3”)-Ib, aph(6)-Id, sul2, tet(A)*
2–460-02_S3_C2	A	O111:H12	3281	IncB/O/K/Z, IncFII	*aatA, aap*	*dfrA14, aph(3”)-Ib, aph(6)-Id, sul2, tet(A)*
2–474-04_S3_C1	A	ONT:H10	43	IncB/O/K/Z, IncFII, IncQ1	*aatA*	*blaTEM-1B, tet(A), dfrA14, dfrA7, sul1, sul2, aph(3”)-Ib, aph(6)-Id*
2–474-04_S3_C2	A	ONT:H10	43	IncB/O/K/Z, IncFII, IncQ1	*aatA, aap*	*blaTEM-1B, tet(A), dfrA14, dfrA7, sul1, sul2, aph(3”)-Ib, aph(6)-Id*
2–474-04_S3_C3	A	ONT:H10	43	IncB/O/K/Z, IncFII, IncQ1	*aatA, aap*	*blaTEM-1B, tet(A), dfrA14, dfrA7, sul1, sul2, aph(3”)-Ib, aph(6)-Id*
3–073-06_S3_C2	A	ONT:H10	43	IncFII, IncQ1	*aatA, aap*	*aph(3”)-Ib, aph(6)-Id, tet(A), sul1, sul2, blaTEM-1B, dfrA7*
7–233-03_S3_C1	A	ONT:H10	10	IncFII	*aap, astA*	-
7–233-03_S3_C3	A	ONT:H10	10	Col, IncFII	*aap*	-
12–05829	B1	O23:H8	26	IncB/O/K/Z, IncFIB, IncFIC	*aatA, aap, astA*	-
381–3	A	O126:H2	10	IncB/O/K/Z	*aatA, aap*	*blaCTX-M-15, blaTEM-1B*
401,368	A	O151:H12	10	Col, IncFIC, IncI1	*aatA, aap, pic, astA*	*dfrA14, aph(3”)-Ib, aph(6)-Id, blaTEM, sul2*
AM22-15AC	A	ONT:H30	3075	ColpVC, IncFII, IncY	*aatA, aap, pet, astA*	*tet(A)*
AM34-8	A	O10:H32	1286	IncFII, IncX1	*aatA, aap, pet*	*blaTEM-1B, qnrS1, tet(A), sul3, aph(6)-Id*
CS01	A	ONT:H30	?	IncB/O/K/Z, IncFIA, IncFIB, IncFII	*aatA, aap*	*dfrA5, tet(B), sul1*
DEC6C	A	O111:H12	10	IncFII	*aatA, aap*	-
ECM-1	A	ONT:H30	2349	IncFIB, IncFII, IncI1, IncX3	*aatA, aap*	*sul2, tet(A), aadA1, dfrA1, qnrB7, qnrS1, qnrS2, blaCTX-M-15, blaTEM*
MRE600	A	O150:H9	?	IncFII	*aatA*	-
NCTC9035	A	O35:H10	10	IncFIC	*aatA, aap, pet, astA*	-
NCTC9062	A	O62:H30	34	IncB/O/K/Z, IncFII	*aatA, aap*	-
NCTC9097	B1	O97:H-	5466	IncFII	*aatA, aap*	-
UPEC-46	A	O116:H12	10	IncB/O/K/Z, IncFII	*aatA, aap, pet, astA*	*aadA1, sul2, tet(B), drfA1*

EAEC genes searched: *aggR* (global virulence regulator), *aatA* (anti-aggregation protein transporter), *aap* (dispersin), *pet* (plasmid-encoded toxin), *pic* (protein involved in colonization) and *astA* (EAEC heat-stable enterotoxin 1); NT, non-typable.

Next, the AFP-positive strains were clustered according to the presence and absence of different virulence factors. The resulting heatmap is presented in Supplementary Figure S2. For this, the presence of 398 genes coding for main virulence-associated factors of IPEC and ExPEC were analyzed. The results are presented in Supplementary Table S6. A high diversity of virulence factors was observed among the AFP-positive *E. coli* strains. All of them harbored widely prevalent genetic determinants of *E. coli*, such as the operons encoding type 1 fimbriae, curli fimbriae, and the Flag-1 flagella system. On the other hand, other important virulence factors, such as iron transport systems (*i.e*., ferric citrate transport, aerobactin, and yersiniabactin), the type 2 secretion system 1 (T2SS-1), type 3 secretion system 2 (ETT-2) and the type 6 secretion systems (T6SS/1, Aai and SCI-I) were not present in all AFP-positive strains. The complete *afp* operon was identified in all 26 strains, and the putative AraC-type regulator, designated as AfpR, was found in all strains (except in *E. coli* 2–474-04_S3_C1) (Supplementary Figure S2).

### AFP is expressed on the bacterial surface of UPEC-46

The AFP mutant and complemented strains were constructed to evaluate the ultrastructure of AFP on the surface of UPEC-46 and its role in bacterial adherence. Initially, the growth and motility of the mutants were analyzed. No marked differences were observed in the generation times for UPEC-46, UPEC-46::*afpA*, and UPEC-46::*afpA* (pPAS3) grown in LB, DMEM, or RPMI (Supplementary Figure S3a). Also, motility was not affected by mutation or complementation of the *afpA* gene (Supplementary Figure S3c).

Next, a specific anti-AFP serum was produced for immunolabeling-based transmission electron microscopy (TEM) analyses. The anti-AFP serum was extensively adsorbed against the *afpA* mutant strain (UPEC-46::*afpA*), and its reactivity was first evaluated by immunoblotting of total protein extract of UPEC-46. As presented in Supplementary Figure S4, the anti-AFP serum recognized a protein band with a molecular weight similar to that predicted for the major pilin AfpA (~21 kDa) in the wild-type but not in the *afpA* mutant strain. Also, the rabbit pre-immune serum did not recognize bands in the protein extracts evaluated (Supplementary Figure S4a).

**Figure 8.**
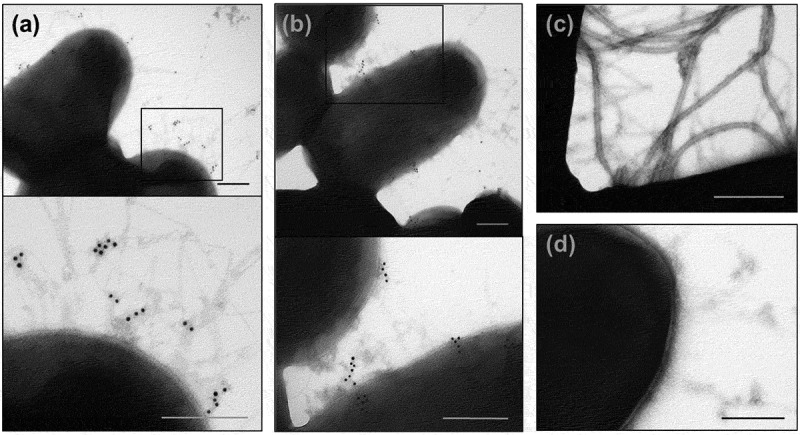
Immunogold labeling of AFP and TEM analysis. (a) wild-type UPEC-46, (b) complemented mutant (UPEC-46::*afpA* (pPAS3)), and (c) *afpA* mutant (UPEC-46::*afpA*) were labeled with adsorbed anti-UPEC-46 serum and goat anti-rabbit IgG conjugated with 10 nm gold particles, contrasted with 2% uranyl acetate in water. (d) Wild-type strain (UPEC-46) labeled with pre-immune serum was used as negative control. Bars = 200 nm

Next, the presence of AFP on the UPEC-46 cell surface was examined by immunogold labeling and electron microscopy, using the pre-immune or the anti-AFP sera as primary antibodies and goat anti-rabbit IgG antiserum conjugated to 10 nm colloidal gold as secondary antibody. Presence of fimbrial structures, presumably pili, were detected with the anti-AFP adsorbed serum in both, wild-type and complemented strains (Figure 8(a,b)) around the bacterial cell. These structures were not observed in the AFP mutant strain (Figure 8(c)), or in wild-type strain treated with the pre-immune serum (negative control of the reaction) (Figure 8(d)).

### AFP contribute to UPEC-46 adherence to both urinary and intestinal epithelial cells

**Figure 9.**
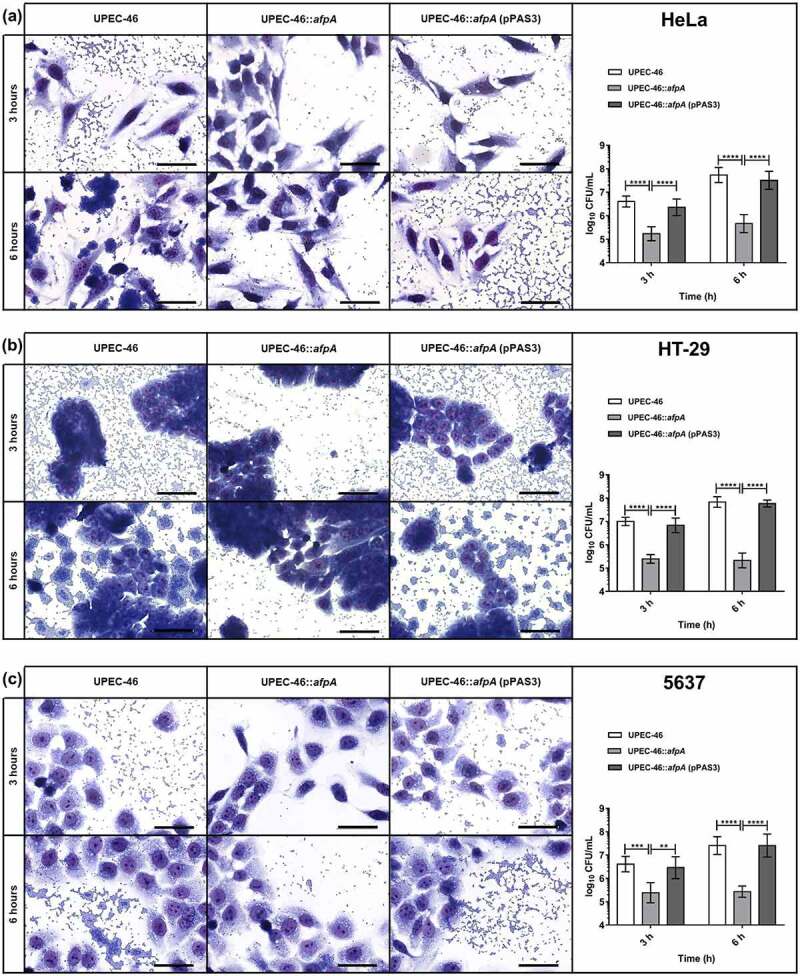
Adherence assays of UPEC-46 and derivatives with different epithelial cell lineages. The adherence ability was identified after 3 h and 6 h of infection, in the presence of 1% D-mannose, using (a) HeLa (human cervical adenocarcinoma), (b) HT-29 (human colon adenocarcinoma), and (c) 5637 (human urinary bladder carcinoma) cells. In qualitative adherence assays, the evaluation of the AA pattern was performed using light microscopy. Bars = 50 µm. For quantitative adherence assays, the number of cell-adhering bacteria was quantified 3 h and 6 h post-infection as described in materials and methods. The adherence assays with UPEC-46, UPEC-46::*afpA*, and UPEC-46::*afpA* (pPAS3) were performed in duplicate and repeated three times. The data presented represent of the mean ± standard deviation. The one-way analysis of variance (ANOVA) followed by Tukey’s multiple-comparison test was used for the statistical analysis. *P-value*: ** *P* < 0.01; *** *P* < 0.001; **** *P* < 0.0001

Qualitative and quantitative adherence assays of UPEC-46, UPEC-46::*afpA*, and UPEC-46::*afpA* (pPAS3) were performed with HeLa, HT-29, and 5637 cells (Figure (9)). In the qualitative assay, the AA pattern displayed by the wild-type strain was abolished in the mutant strain (UPEC-46::*afpA*) and restored in the complemented strain (UPEC-46::*afpA* (pPAS3)) with all three cell lines.

In the quantitative adherence assays, UPEC-46 showed a strong adherence to all eukaryotic cell lines upon different time points analyzed (3 and 6 h), and UPEC-46::*afpA* exhibited a significant (p < 0.01) decrease in adherence to all cell lines. Complementation of UPEC-46::*afpA* restored the adherence score under all conditions tested. In summary, these assays showed that AFP contributes to the adherence phenotype of UPEC-46 and to the establishment of the AA pattern.

## Discussion

In this study, we investigated the geno- and phenotypic characteristics of UPEC-46. In our initial analyses, the characteristic AA pattern was also observed with intestinal (HT-29) and bladder (5637) cell lines. These results suggested that UPEC-46 could be a hybrid-pathogenic (UPEC/EAEC) strain able to cause intestinal and extraintestinal infections. In this context, the absence of specific virulence markers used to define ExPEC or UPEC intrinsic virulence of an *E. coli* strain [27,77] classified UPEC-46 as ExPEC negative (ExPEC^−^) and UPEC negative (UPEC^−^). Although the presence of these specific virulence markers correlates with the extraintestinal and uropathogenic potential of *E. coli* isolates, extraintestinal infections may nevertheless be caused by strains devoid of these genes [29,33,36,37,81,82].

The genomic analysis of UPEC-46 identified several virulence genes, including those encoding adhesins (*e.g.*, operons *sfm, yad, ybg, ycb, yde, yeh, yfc, yhc*, and *yra*), bacteriocins (*colE1* and operon *mcb*), iron acquisition factors (*fyuA*), polysaccharide capsule (group 4 capsule), serum resistance (*iss*), and invasins (*ibeB* and *ibeC*), some of them being epidemiologically associated with extraintestinal infections [25,83–86]. In addition, like other hybrid *E. coli* strains (UPEC/EAEC), UPEC-46 carries virulence genes related to the EAEC pathotype (*e.g., aap, pet*, and the *aai* and *aat* operons), reflecting the dual genotypic profile commonly associated with these strains.

Secretion of bacteriocins increases the competitiveness of *E. coli* strains [87]. Colicin E1 is a bacteriocin that has been associated as an important virulence factor in UPEC [88]. As UPEC-46 harbored *colE1* and produced colicin E1 *in vitro*, this strain has a competitive colonizing potential in the urinary and intestinal tracts since bacteriocin production may interfere with the growth of colicin-sensitive *E. coli* variants.

Pet is a toxin firstly described in EAEC [15] that causes intracellular cleavage of fodrin, disrupting the actin cytoskeleton, leading to enterotoxic and cytotoxic effects [16,89]. The intrinsic function of Pet is associated with EAEC intestinal infections [90]. However, some studies showed the presence of the *pet* gene in ExPEC strains [29,32]. In addition, our group verified that Pet has a cytotoxic effect on the human urinary bladder epithelial cell line 5637 (unpublished data). As Pet secretion was detected in the culture supernatant of UPEC-46, this toxin could have a role in uropathogenesis.

UPEC-46 adhered to different epithelial cell lines in the AA pattern and expressed important virulence factors associated with biofilm formation, such as type 1 fimbriae, curli, and cellulose; however, it was unable to form strong biofilms on different abiotic surfaces and under different culture conditions, including growth in pooled human urine. Some studies have shown that specific EAEC virulence factors, such as *aggR* (transcriptional activator) and AAFs (EAEC fimbriae), are involved in biofilm formation at levels significantly higher than strains negative or defective of these factors [38,47], corroborating the results obtained in this study, since UPEC-46 is devoid of these two virulence factors.

The genomic analysis of UPEC-46 showed that this strain carries three distinct plasmids, two of which (p46-2 and p46-3) were successfully transferred by conjugation, thereby allowing the identification of a correlation of p46-2 with antimicrobial resistance. The genotypic analyses identified the *pil*/*tra* operons and *mbeA* and *mbeC* genes related to plasmid transfer in these two plasmids. In addition, it was observed that p46-1 carries some EAEC virulence genes (e.g., *aatA, pet, aap*, and the *aai* operon). However, it is devoid of *aggR*, which encodes the EAEC master virulence regulator [20]. As EAEC strains may be classified as ”typical“ or ”atypical“, based on the presence or absence of *aggR*, respectively [91], the presence of p46-1 provides to UPEC-46 a virulence profile of atypical EAEC strains. Also, p46-1 presented some similarities to the pAFP plasmid described by Lang et al. [78], harboring the *afp* and *aat* operons, *aap, afpR*, and parts of the *aai* operon.

In this context, from the data observed by the geno- and phenotypic analysis of UPEC-46, two hypotheses were raised regarding its phylogenetic origin: UPEC-46 could be an atypical EAEC strain that, over time, acquired ExPEC genes therefore enabling UPEC-46 to establish UTI. Alternatively, an ExPEC strain could have received the plasmid p46-1 (EAEC-associated plasmid). Our phylogenetic analyses indicated that UPEC-46 was placed in a separate phylogenetic branch together with other strains with an atypical EAEC genomic background that have been isolated from patients with or without diarrhea. Interestingly, analyzing different ST10 *E. coli* strains, we showed that UPEC-46 belongs to a specific cluster of atypical EAEC strains, presenting the *afp* operon genes and its regulator gene (*afpR*). In UPEC-46, the *afp* operon and the *afpR* regulatory gene were identified in the plasmid p46-1, along with other EAEC-associated genes. Thus, the data obtained from our phylogenetic analysis indicate that UPEC-46 has a genomic background associated with atypical EAEC strains harboring the *afp* operon. AFP was described to confer the AA pattern of a Shiga toxin-producing *E. coli* (STEC)/EAEC strain of serotype O23:H8 [78], and was recently found in atypical EAEC strains isolated from diarrhea [80]. Therefore, we decided to investigate further the role of AFP in adherence traits of UPEC-46.

AFP presentation was observed by immunogold labeling on the surface of UPEC-46 as fimbrial structures not assembled in bundles, as described for the bundle-forming pilus (BFP) of enteropathogenic *E. coli*, another type 4 pili [92]. It is interesting to note that the operons encoding AFP and BFP share the same genetic organization but have only approximately 42% protein identity [78]. The ultrastructure of AFP observed by TEM on the surface of UPEC-46 resembles fimbria-like structures observed by scanning electron microscopy with the STEC/EAEC strain as described by Lang et al. [78]. The reason for the different appearance of AFP and BFP is unclear and may be due to differences in the amino acid composition of both pilin subunits. The presence of non-labeled fimbrial structures on the bacterial surface observed in TEM preparations supports the idea that other adhesins could be co-expressed together with AFP. Furthermore, the amount of fimbrial structures labeled with anti-AFP serum could be also related to the culture medium (LB medium) and other bacterial growth conditions used in this study. Therefore, future studies will be needed to verify the best condition for AFP expression in *E. coli* strains *in vitro*.

Our bioinformatic analyses showed that AFP-positive isolates do not share a common profile of virulence factors. The diversity of virulence profiles among *E. coli* strains classified by isolation site or classical genetic defining markers is a common feature resulting from the high frequency of horizontal gene transfer of *E. coli* virulence genes. Thus, the AFP-positive strains cannot be associated with a single virulence profile. However, the results obtained in this study show that the absence of specific genes associated with ExPEC and the presence of atypical EAEC determinants could be associated with the genotypic characteristics of these strains.

The absence of genes encoding typical ExPEC adhesins or aggregative adherence fimbriae (AAF) characteristic of EAEC in the genome of UPEC-46 supports the idea that other adhesins could be involved in establishing the AA phenotype of this strain. Our data suggested that AFP mediate the AA pattern of UPEC-46. Therefore, we analyzed the effects of *afpA* mutation in UPEC-46 to evaluate the role of AFP in its adherence phenotype. A significant reduction of the adherence and absence of the AA pattern was observed on HeLa, HT-29, and 5637 cells. The effect of the *afpA* mutation confirmed that the novel AFP adhesin is essential for adhesion and the establishment of the AA pattern, as previously shown for STEC/EAEC and EAEC strains [78]. Although, to our knowledge, there are still no studies showing the prevalence of AFP in collections of *E. coli* strains isolated from UTI, genes of the *afp* operon were detected in atypical EAEC strains obtained from diarrheal patients in Brazil [80]. Further investigations by our group are currently in progress to examine whether AFP is involved in the uropathogenicity of the UPEC-46 strain and whether this adhesin also plays a role in intestinal colonization. We also highlight that the present study represents the first report that shows the role of AFP in establishing the AA pattern on either bladder or intestinal epithelial cells (HT-29 and 5637 lineages) and characterizes the ultrastructure of AFP.

In conclusion, the emergence of novel *E. coli* variants resulting from the combination of multiple traits of already known pathotypes represents a new challenge in epidemiology and pathogenesis. In this aspect, although isolated from UTI, UPEC-46 presented important characteristics associated with atypical EAEC allowing its classification as a hybrid UPEC/EAEC strain. We also showed that AFP is essential for the AA phenotype on bladder and colorectal epithelial cells. However, its relationship with uropathogenesis and intestinal colonization needs to be better understood. Thus, future studies will be required to understand the prevalence of these hybrid-pathogenic *E. coli* strains and characterize in-depth the molecular basis associated with UTI.

## Supplementary Material

Supplemental Material

## Data Availability

The authors confirm that the data supporting the findings of this study are available within the article and its supplementary materials. The NCBI accession numbers of genome sequences are PRJNA728080, JAHBCK000000000, NZ_JAHBCK010000003, NZ_JAHBCK010000004, and NZ_JAHBCK010000007, found in https://www.ncbi.nlm.nih.gov/. The raw data used to generate graphs and phylogenetic analyses are available in the Butantan Institute Repository [https://repositorio.butantan.gov.br/handle/butantan/3890].
