# Supplementary material for: The aggregate-forming pili (AFP) mediates the aggregative adherence of a hybrid-pathogenic Escherichia coli (UPEC/EAEC) isolated from a urinary tract infection
Source: Virulence. 2021 Dec 20;12(1):3073–93. doi: 10.1080/21505594.2021.2007645 (PMC8923075; doi:10.1080/21505594.2021.2007645)
Supplement: Supplemental Material [file KVIR_A_2007645_SM6615.zip › supplementary/Suppl. Figure 1.docx]

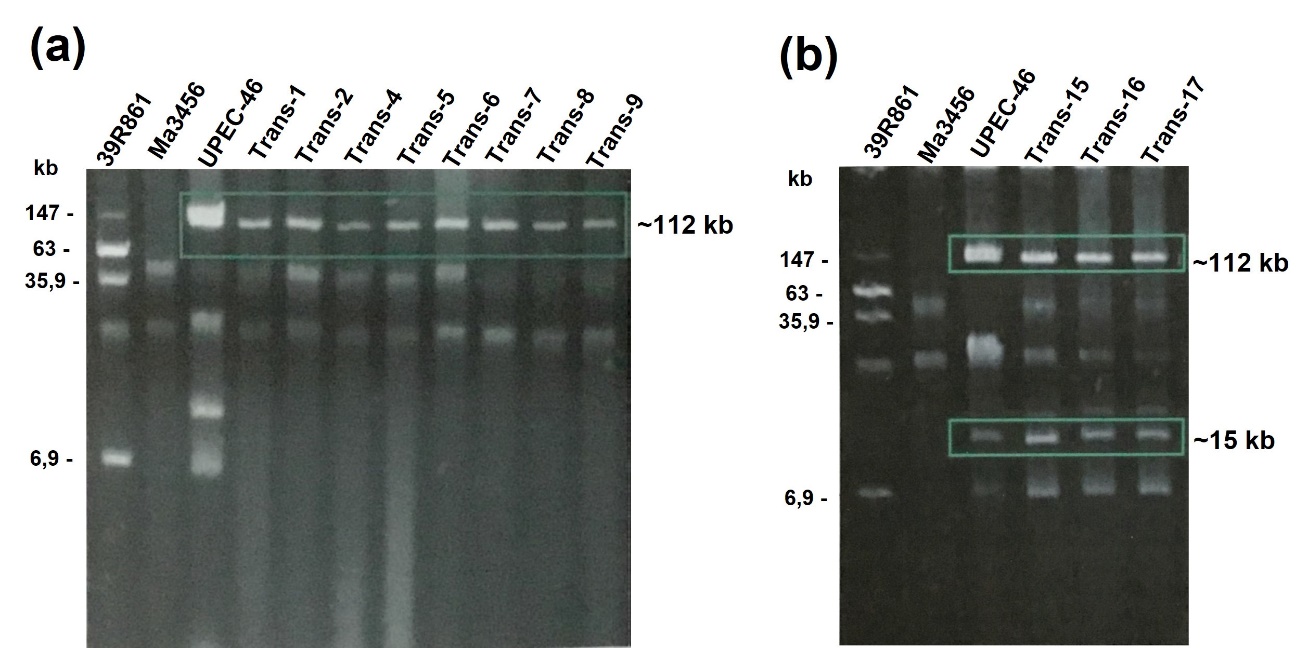


**Supplementary Figure 1.** **Analysis of the conjugative plasmids present in the UPEC-46 strain**. **(a)** Plasmid profile of the transconjugant strains (Trans 1-9) carrying only the ~112 kb plasmid. **(b)** Analysis of plasmid profile of the transconjugant strains (Trans 15-17) carrying the ~112 kb and ~15 kb plasmids. The plasmid content of different strains analyzed was obtained by alkaline extraction, followed by electrophoresis in 0.8% agarose gel in TBE buffer. Approximate sizes were predicted based on the plasmid migration in agarose gel. The UPEC-46 strain was used as a donor and *E. coli* strain MA3456 as a recipient. The *E. coli* strain 39R861 represents the standard strain containing plasmids of known molecular weights.
