# Supplementary material for: The aggregate-forming pili (AFP) mediates the aggregative adherence of a hybrid-pathogenic Escherichia coli (UPEC/EAEC) isolated from a urinary tract infection
Source: Virulence. 2021 Dec 20;12(1):3073–93. doi: 10.1080/21505594.2021.2007645 (PMC8923075; doi:10.1080/21505594.2021.2007645)
Supplement: Supplemental Material [file KVIR_A_2007645_SM6615.zip › supplementary/Suppl. Figure 2.docx]

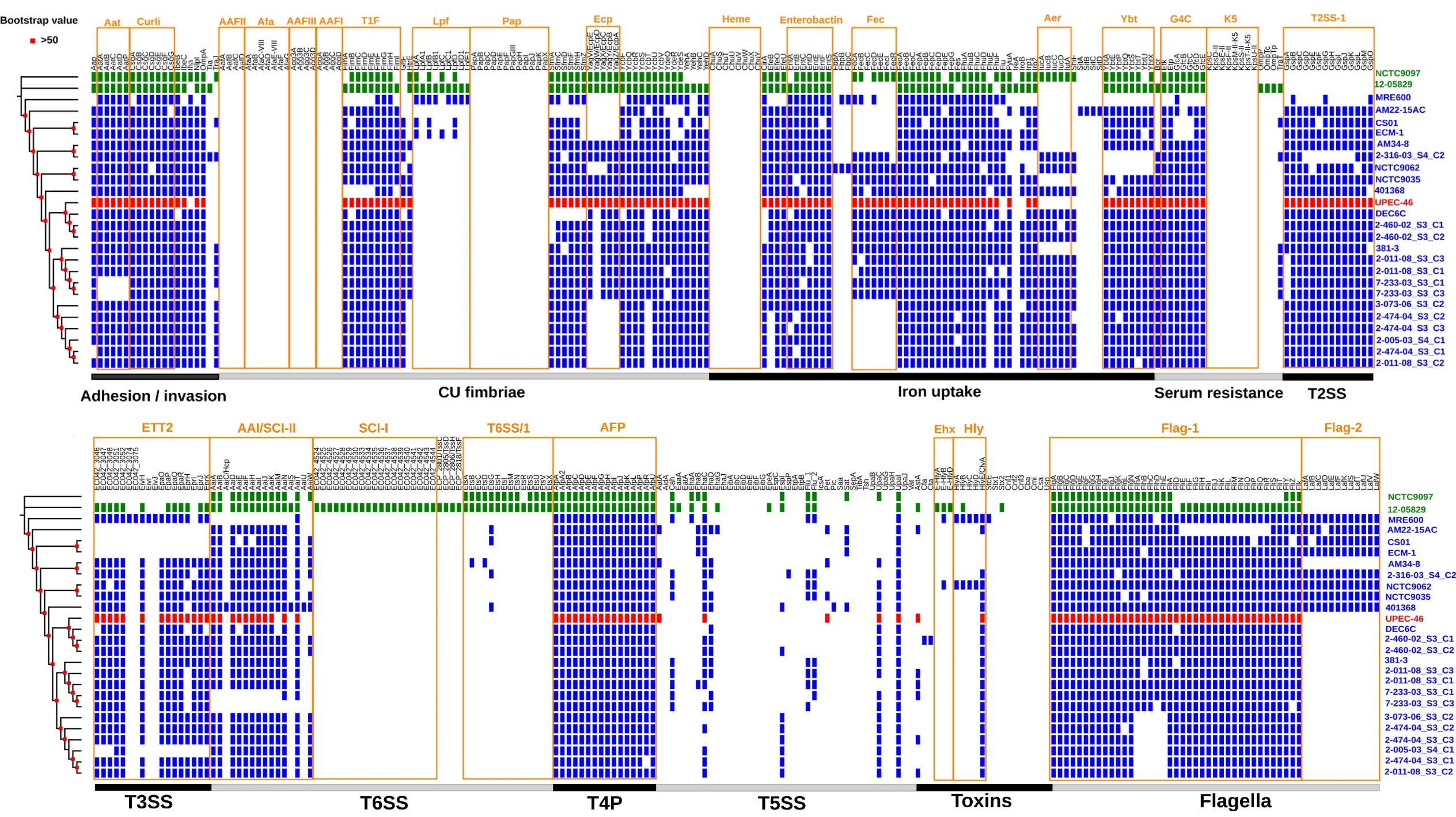


**Supplementary Figure 2.** **Heatmap indicating presence or absence of virulence factors**. Each row of the binary matrix indicates the presence or absence of a virulence-associated gene (a BLASTP+ hit). Virulence factor classes are indicated at the side in black and grey. Strain names are color-coded for phylogroup affiliation (green: B1, blue: A). UPEC-46 is shown in red. Well-known *E. coli* virulence determinants are indicated by orange boxes. The clustering dendrogram attached to the heatmaps is based on the whole binary dataset of a best-scoring ML tree with 1000 bootstrap resampling. Bootstrap support value is arbitrarily indicated at the bifurcations of the cladogram. T2SS, type 2 secretion system; T3SS, type 3 secretion system; T4P, type 4 pili; T5SS, type 5 secretion system; T6SS, type 6 secretion system; CU fimbriae, chaperone-usher fimbriae.
