# Supplementary material for: The aggregate-forming pili (AFP) mediates the aggregative adherence of a hybrid-pathogenic Escherichia coli (UPEC/EAEC) isolated from a urinary tract infection
Source: Virulence. 2021 Dec 20;12(1):3073–93. doi: 10.1080/21505594.2021.2007645 (PMC8923075; doi:10.1080/21505594.2021.2007645)
Supplement: Supplemental Material [file KVIR_A_2007645_SM6615.zip › supplementary/Suppl. Figure 3.docx]

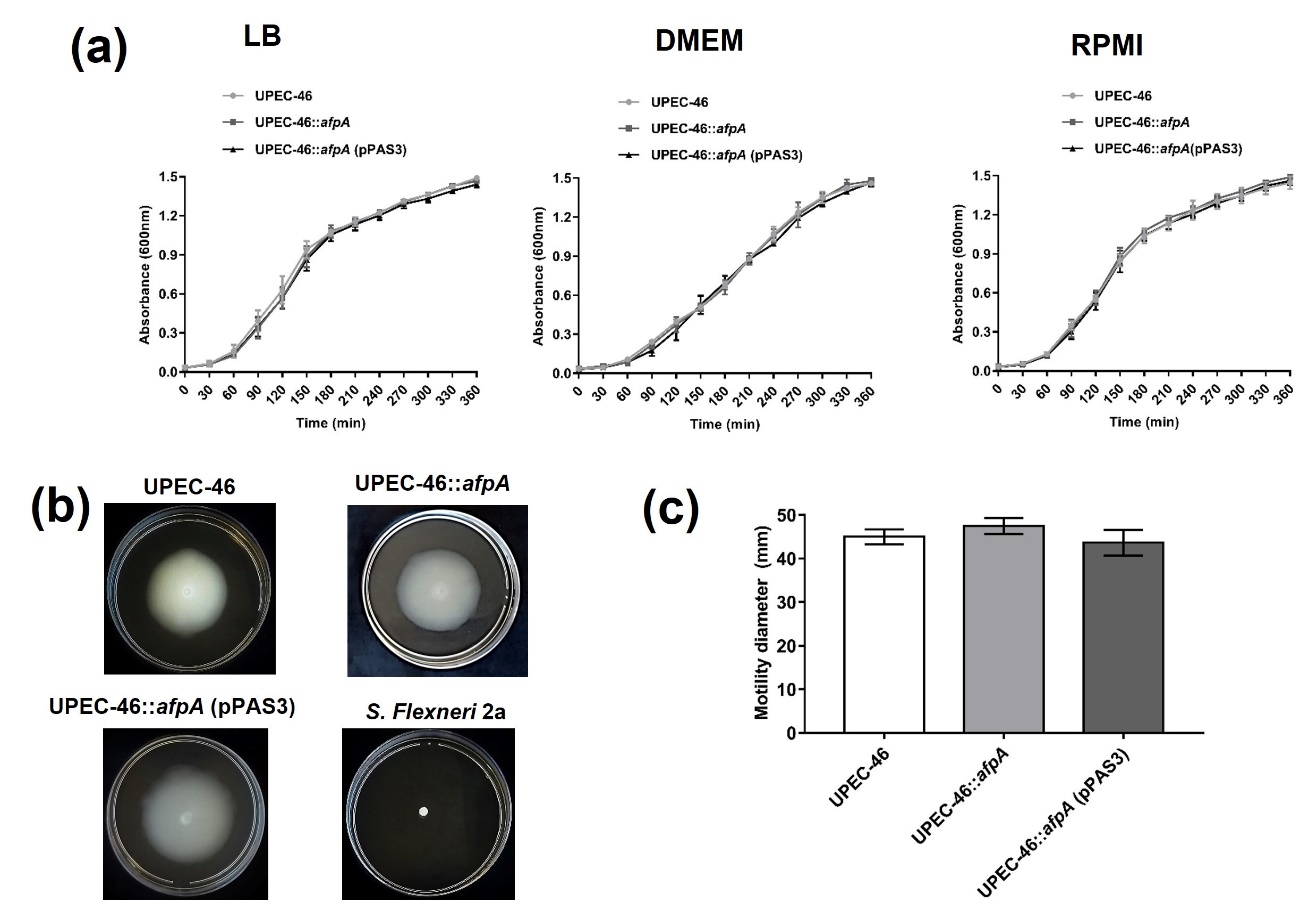


**Supplementary Figure 3. Phenotypic characteristics of UPEC-46 and derivatives. (a)** Growth curve of UPEC-46 and derivatives in different culture media. The growth curve of UPEC-46, UPEC-46::*afpA*, and UPEC-46::*afpA* (pPAS3) were tested using the following culture media: LB, DMEM, and RPMI. Absorbance was measured every 30 min for 6 h at 600 nm. The tests were carried out on three different days. The data presented consist of the mean ± Standard Deviation. LB, lysogeny broth; DMEM, Dulbecco modified Eagle medium; RPMI, Roswell Park Memorial Institute medium. **(b)** Motility of UPEC-46 and derivatives in motility agar (LB and agar 0.3%). *S. flexneri* 2a was used as a negative control. **(c)** Motility of UPEC-46 and derivatives in motility agar. The assay was performed in duplicate and repeated three times. The data presented consist of the mean ± Standard Deviation. The one-way analysis of variance (ANOVA) followed by Dunnett’s multiple-comparison test was used to see differences between UPEC-46 and construction strains. For motility assays, the strains were cultured in motility agar at 37 ºC during 18 h.
