# Supplementary material for: The aggregate-forming pili (AFP) mediates the aggregative adherence of a hybrid-pathogenic Escherichia coli (UPEC/EAEC) isolated from a urinary tract infection
Source: Virulence. 2021 Dec 20;12(1):3073–93. doi: 10.1080/21505594.2021.2007645 (PMC8923075; doi:10.1080/21505594.2021.2007645)
Supplement: Supplemental Material [file KVIR_A_2007645_SM6615.zip › supplementary/Suppl. Figure 4.docx]

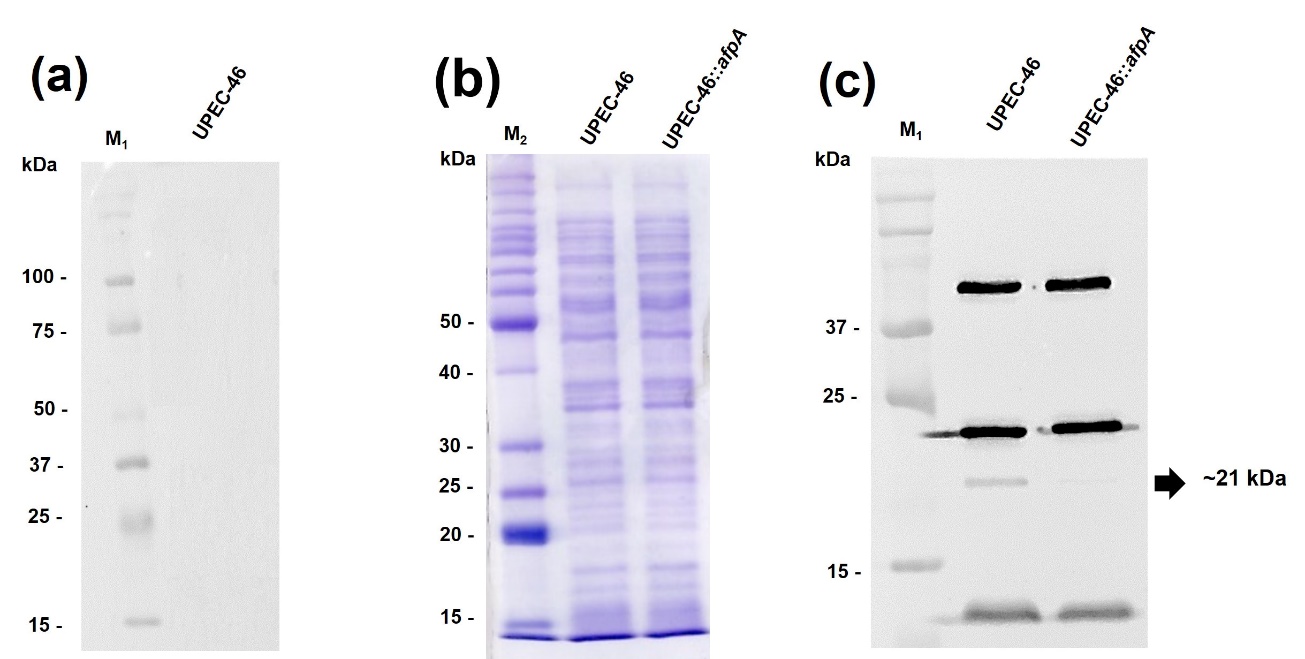


**Supplementary Figure 4.** **Analyses of anti-UPEC-46 serum by immunoblotting. (a)** Wild-type strain (UPEC-46) incubated with pre-immune serum (negative control). **(b)** 12% SDS-polyacrylamide gel of cell lysed proteins after Coomassie Brilliant Blue staining. **(c)** Immunoblotting of cell lysed proteins using the adsorbed anti-UPEC-46 serum. M: molecular weight; M_1_: Precision Plus Protein™ Kaleidoscope Standards (Bio-Rad).; M_2_: BenchMark Protein Ladder (Thermo).
