# Supplementary material for: The aggregate-forming pili (AFP) mediates the aggregative adherence of a hybrid-pathogenic Escherichia coli (UPEC/EAEC) isolated from a urinary tract infection
Source: Virulence. 2021 Dec 20;12(1):3073–93. doi: 10.1080/21505594.2021.2007645 (PMC8923075; doi:10.1080/21505594.2021.2007645)
Supplement: Supplemental Material [file KVIR_A_2007645_SM6615.zip › supplementary/Suppl._Table_1.docx]

**Supplementary Table 1.** Strains and plasmids used in this study.

| Strains/plasmids | Characteristics | Reference |
| --- | --- | --- |
| Strains | | |
| UPEC-46 | *E. coli* wild-type strain, O166:H12 | Abe at al. (2008) |
| UPEC-46::*afpA* | UPEC-46 harboring pPAS2 integrated into the *afpA* gene | This work |
| UPEC-46::*afpA* (pPAS3) | UPEC-46 harboring pPAS2 integrated into the *afpA* gene and complemented with pPAS3 | This work |
| *E. coli* DH5α | K-12 *E. coli* | Stratagene, USA |
| S17-1(λpir) | Conjugative K-12 lysogenized for the *pir* gene, which permits replication of R6K plasmid replicons | Simon et al. (1983) |
| *E. coli* DH5α (pPAS1) | DH5α strain carrying pPAS1 plasmid | This work |
| *E. coli* S17-1(λpir) (pPAS2) | S17-1(λpir) strain carrying pPAS2 plasmid | This work |
| *E. coli* DH5α (pPAS3) | DH5α strain carrying pPAS3 plasmid | This work |
| *E. coli* ATCC 25922 | *E. coli* reference strain for quality control used in antibiotic susceptibility test | CLSI (2017) |
| *E. coli* 042 | EAEC strain (O44:H18), positive control for Pet production and biofilm formation | Nataro et al. (1995) |
| *E. coli* C600 | *E. coli* strain used as an indicator for bacteriocins production | Appleyard (1954) |
| *E. coli* E2348/69 | Enteropathogenic strain (O127:H6), positive control for curli expression | Levine et al. (1978) |
| *E. coli* BA320 | Enteropathogenic strain (O55:H7), positive control for cellulose expression | Bueris et al. (2007) |
| E. coli BA732 | EAEC strain (O17:H18), negative control for Pet production | Bueris et al. (2007) |
| *E. coli* 39R861 | *E. coli* strain used as reference for the plasmid profile | Moran et al. (2019) |
| *E. coli* MA3456 | *E. coli* donor strain used in conjugation experiment | Lima et al. (2019) |
| *S. flexneri* 2a | *Shigella flexneri* strain used as negative control for motility test | Bârzu et al. (1997) |
| Plasmids | | |
| pGEM-T Easy | LacZ cloning vector (Ap^r^) | Promega, USA |
| pPAS1 | *afpA* gene fragment, cloned into pGEM T-easy (Ap^r^) | This work |
| pJP5603 | R6K suicide plasmid (Kn^r^) | Penfold and Pemberton, 1992 |
| pPAS2 | *afpA* gene fragment, digested with *EcoRI*, cloned into pJP5603 (Kn^r^) | This work |
| pACYC177 | Small-copy number expression vector permitting expression of foreign genes (Ap^r^ Kn^r^) | Chang and Cohen (1978) |
| pPAS3 | *afpA* gene cloned into pACYC177 to permit expression of AfpA (Ap^r^) | This work |

Kn^r^, kanamycin resistant; Ap^r^, ampicillin resistant.

**REFERENCES**

Abe CM, Salvador FA, Falsetti IN, et al. Uropathogenic *Escherichia coli* (UPEC) strains may carry virulence properties of diarrhoeagenic *E. coli*. *FEMS Immunol Med Microbiol*. 2008;52(3):397-406. doi:10.1111/j.1574-695X.2008.00388.x

Appleyard RK. Segregation of new lysogenic types during growth of a doubly lysogenic strain derived from *Escherichia coli* K12. *Genetics*. 1954;39(4):440-452.

Bârzu S, Benjelloun-Touimi Z, Phalipon A, et al. Functional analysis of the *Shigella flexneri* IpaC invasin by insertional mutagenesis. *Infect Immun*. 1997;65(5):1599-1605. doi:10.1128/iai.65.5.1599-1605.1997

Bueris V, Sircili MP, Taddei CR, et al. Detection of diarrheagenic *Escherichia coli* from children with and without diarrhea in Salvador, Bahia, Brazil. *Mem Inst Oswaldo Cruz*. 2007;102(7):839-844. doi:10.1590/s0074-02762007005000116

Chang AC, Cohen SN. Construction and characterization of amplifiable multicopy DNA cloning vehicles derived from the P15A cryptic miniplasmid. *J Bacteriol*. 1978;134(3):1141-1156. doi:10.1128/jb.134.3.1141-1156.1978

Clinical and Laboratory Standards Institute (CLSI). Performance standards for antimicrobial susceptibility testing. 27th ed. Tertel ML, Christopher JP, Martin L & Russell MA, eds. Wayne, PA;2017.

Levine MM, Bergquist EJ, Nalin DR, et al. *Escherichia coli* strains that cause diarrhoea but do not produce heat-labile or heat-stable enterotoxins and are non-invasive. *Lancet*. 1978;1(8074):1119-1122. doi:10.1016/s0140-6736(78)90299-4

Lima MP, Yamamoto D, Santos ACM, et al. Phenotypic characterization and virulence-related properties of *Escherichia albertii* strains isolated from children with diarrhea in Brazil. *Pathog Dis*. 2019;77(2):ftz014. doi:10.1093/femspd/ftz014

Moran RA, Anantham S, Hall RM. An improved plasmid size standard, 39R861. *Plasmid*. 2019;102:6-9. doi:10.1016/j.plasmid.2019.01.002

Nataro JP, Deng Y, Cookson S, et al. Heterogeneity of enteroaggregative *Escherichia coli* virulence demonstrated in volunteers. *J Infect Dis*. 1995;171(2):465-468. doi:10.1093/infdis/171.2.465

Penfold RJ, Pemberton JM. An improved suicide vector for construction of chromosomal insertion mutations in bacteria. *Gene*. 1992;118(1):145-146. doi:10.1016/0378-1119(92)90263-o

Simon R, Priefer U, Pühler, AA. Broad host range mobilization system for *in vivo* genetic engineering: transposon mutagenesis in Gram negative bacteria. *Nat Biotechnol.* 1983;1:784–791. doi:10.1038/nbt1183-784
